# Genomic structure, ecological suitability and biogeographic history of the palm *Acrocomia aculeata* across Central America

**DOI:** 10.3389/fpls.2025.1724384

**Published:** 2026-01-27

**Authors:** Jonathan A. Morales-Marroquín, Erick René López de Paz, Rocío Silva-Rivera, Ana Flávia Francisconi, Roger Alejandro Orellana-Hernandez, José M. Palacios, Emmanuel Araya-Valverde, Elizabeth Arnáez−Serrano, João Victor da Silva Rabelo-Araujo, Caroline Bertocco Garcia, Matheus Scaketti, Carlos A. Colombo, Brenda Gabriela Díaz-Hernández, José Baldin Pinheiro, Maria Imaculada Zucchi

**Affiliations:** 1 Department of Genetics, ”Luiz de Queiroz“ College of Agriculture, University of São Paulo (USP Esalq), Piracicaba, São Paulo, Brazil; 2 Centro de Estudios Ambientales y Biodiversidad, Universidad del Valle de Guatemala, Guatemala, Guatemala; 3 Hylos, Guatemala; 4 Escuela de Biología, Facultad de Ciencias Químicas y Farmacia, Universidad de San Carlos de Guatemala (USAC), Guatemala, Guatemala; 5 Servicio Nacional de Sanidad e Inocuidad Agroalimentaria (SENASA), Secretaría de Agricultura y Ganadería (SAG), Tegucigalpa, Honduras; 6 Department of Botany, University of Panama, Panama, Panama; 7 Centro Nacional de Innovaciones Biotecnológicas (CENIBiot), Centro Nacional de Alta Tecnología – Consejo Nacional de Rectores (CeNAT-CONARE), San José, Costa Rica; 8 Escuela de Biología, Instituto Tecnológico de Costa Rica (TEC), Cartago, Costa Rica; 9 Genetics and Molecular Biology Department, Biology Institute, University of Campinas (UNICAMP), Campinas, Brazil; 10 Institute of Agronomy (IAC), Research Center of Plant Genetic Resources, Campinas, São Paulo, Brazil; 11 Unidade Regional de Pesquisa e Desenvolvimento (APTA), Piracicaba, São Paulo, Brazil

**Keywords:** allelic richness, *Arecaceae*, Coyol, ecological niche modelling, GBS, Macaúba, population genetics, SNP

## Abstract

Central America is an understudied global hotspot of plant biodiversity and harbors *Acrocomia aculeata* (Coyol or Macaúba), a neotropical palm with significant potential for oil and biofuel production. Historically, the region has functioned as a biogeographic land bridge, an isthmus, connecting North and South American biota. Here, we investigate how genomic diversity and potential distribution patterns of *A. aculeata* are shaped across Central America. A total of 259 samples were collected from Guatemala, Honduras, Nicaragua, Costa Rica, and Panama, covering the full extent of the Central American isthmus. Using a double-digest genotyping-by-sequencing (ddGBS) approach and ecological niche modeling, we assessed variation at 1,523 single nucleotide polymorphisms (SNPs) and evaluated environmental suitability across the region. Our analyses reveal three major genomic clusters: Mesoamerican, Costa Rican, and Panamanian, each comprising subpopulations with distinct levels of genetic diversity. The Mesoamerican group (Guatemala, Honduras, and northern Nicaragua) exhibited the highest diversity and unique genetic signatures, likely reflecting historical migrations and acting as a biodiversity cradle during periods when southern portions of the isthmus were submerged. Biogeographic features such as the Nicaraguan Depression and the Talamanca Cordillera contributed to regional genetic differentiation. Ecological niche models identified Central American pacific lowlands, forested areas, rangelands, and agroecosystems as suitable habitats for *A. aculeata*. Our combined results reflect the evolutionary history and population structure of *A. aculeata* in Central America, highlighting the influence of South American source populations and regional barriers. These findings provide a critical foundation for conservation and breeding programs aiming to preserve the genetic diversity and adaptive potential of *A. aculeata* in a rapidly changing and neglected biodiversity hotspot.

## Introduction

Central America is one of the world’s major plant diversity hotspots and provides a unique natural laboratory for studying how geological history and environmental heterogeneity shape tropical plant evolution. Palms, in particular, are excellent models for biogeographic and genomic studies due to their broad ecological amplitude, long lifespans, and deep associations with both natural and human-modified landscapes (Couvreur et al., 2011; Cámara-Leret et al., 2014; Eiserhardt et al., 2017). Among them, *Acrocomia aculeata* (Jacq.) Lodd. ex Mart. (Bactridinae subtribe of Arecaceae) commonly known as Coyol, Corozo, or Macaúba, stands out as a widespread, open-canopy palm of ecological, cultural, and economic importance. It is a high-oil-yielding species with a long history of human use in Mesoamerica and occurs across diverse tropical biomes from Mexico to Brazil (Díaz et al., 2021; Morales-Marroquín et al., 2025). Its fruits and byproducts have multiple uses, from biofuel and food to cosmetics, making it a promising crop for sustainable agriculture and regional bioeconomies. Its adaptability to ecotones and human-modified landscapes further supports its potential for integration into agroecological and restoration systems (Vargas-Carpintero et al., 2021).

Across its range, *A. aculeata* occupies transitional zones between humid and dry ecoregions and is divided into two broad genomic lineages: a Central American group and a South American group (Díaz et al., 2021; Morales-Marroquín et al., 2025). *A. aculeata* exhibits a mixed mating system with a predominance of outcrossing in South American populations (Scariot et al., 1995; Abreu et al., 2012; Lanes et al., 2016; Díaz-Hernández et al., 2023). This high outcrossing tendency is linked to floral protogyny, where stigmas are receptive before pollen release, reducing self-fertilization and promoting cross-pollination (Scariot et al., 1991). Pollination is mainly mediated by beetles and weevils, while seeds are dispersed primarily by gravity and occasionally by cattle and humans, leading to local spatial genetic structure (Abreu et al., 2012; Göldel et al., 2016; Carreño-Barrera et al., 2025). These reproductive traits facilitate gene flow within continuous populations but may limit connectivity in fragmented landscapes, as observed in the present study.

The geological evolution of the Central American Isthmus, a narrow strip of land connecting two larger landmasses and separating two major bodies of water, has been central in shaping Neotropical biodiversity. This land bridge emerged through the accretion of the Maya, Chortis, Chorotega, and Chocó tectonic blocks and served both as a corridor and a barrier for biotic exchange during the Miocene–Pliocene (Bagley and Johnson, 2014; Bacon et al., 2015). These geological transitions structured connectivity in many plant lineages and likely influenced the northward migration and diversification of *Acrocomia*. Cultural processes further shaped its distribution, as pre-Columbian societies used the species for food and ritual purposes for at least 4,000 years (Lentz, 1990; Clement, 1999; Ramírez-Núñez et al., 2019). Recent research has shown that native Central American palm lineages, including *A. aculeata*, often reflect *in situ* diversification rather than recent colonization, suggesting a long-standing evolutionary history within the region (Cano et al., 2022).

Despite its ecological and economic importance, the Central American *A. aculeata* gene pool has never been genomically characterized. This gap is part of a broader pattern of scientific underrepresentation in Central America, one of the most neglected tropical biodiversity hotspots, where genomic data for native plants remain scarce (Rydén et al., 2020; Morales-Marroquín et al., 2022). Addressing these gaps is essential for uncovering novel genetic resources, informing conservation priorities, and promoting sustainable agricultural and ecological practices in a region increasingly affected by climate change.

Here, we combine genome-wide SNPs generated through ddGBS with ecological niche modeling to characterize the genomic structure and climatic suitability of *A. aculeata* across the Central American Isthmus. We test three key hypotheses: (1) that major geological features, particularly the Nicaraguan Depression and the Talamanca Cordillera, have shaped population structure; (2) that contemporary environmental and land-use gradients further reinforce genetic differentiation; and (3) that Central American populations represent a distinct northern lineage relative to South American *A. aculeata*. By addressing these questions, our study fills a critical gap in the understanding of *A. aculeata* evolutionary history and provides essential genomic information for conservation, domestication, and sustainable use of this emerging tropical crop.

## Materials and methods

### Plant material and DNA isolation

To characterize the genetic structure of *A. aculeata* across Central America, we first analyzed a dataset containing all samples collected across the Central American Isthmus (Guatemala, Honduras, Nicaragua, Costa Rica, and Panama). This dataset enabled us to evaluate genomic diversity, population structure, and regional differentiation. To place these patterns in a broader biogeographic context and test whether the Central American gene pool is distinct from the South American lineage, we generated a comparative sub-dataset including samples from Amazonas, Roraima, Minas Gerais, and São Paulo, as well as individuals of the sister species *A. intumescens* and *A. totai*. This allowed us to assess the genomic distinctiveness of the Central American gene pool and determine whether it forms a cohesive lineage separate from South American populations.

**Figure 1:**
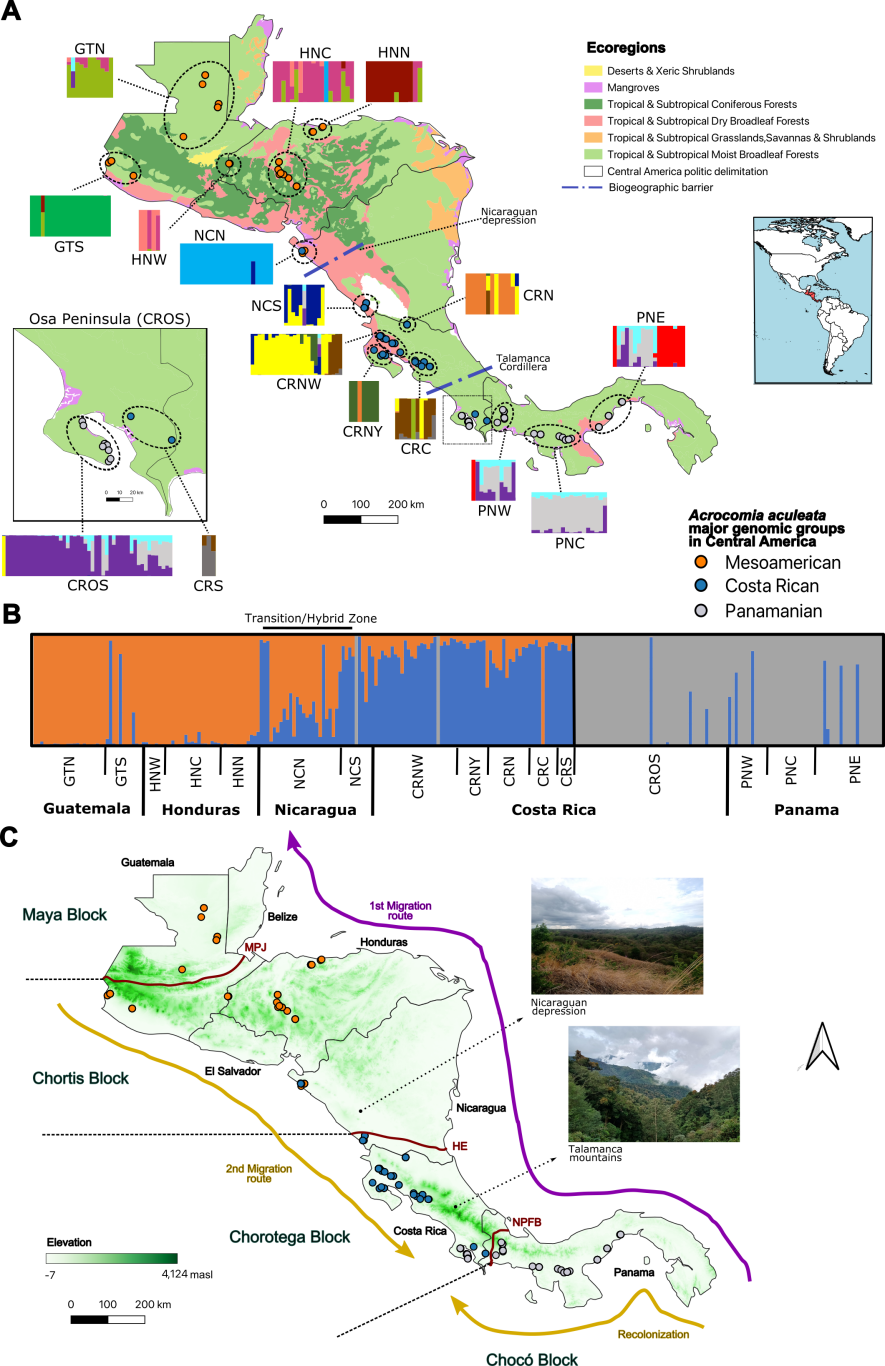
Map of sampling locations for *Acrocomia aculeata* along with major genetic divergences among Central America suggested by DAPC. **(A)** Central America Map showing ecoregions, each bar plot in every subpopulation shows the genetic clusters represented by different colors. The color of each population point corresponds to the major genomic group. **(B)** Bar plot showing the 3 major genetic clusters within subpopulations in Central America based on DAPC. Acronyms follow **Table 1** Sub-regions. **(C)** Tectonic blocks of Central America along with biogeographic barriers. Tectonic limits: MPJ: Motagua–Polochic–Jocotán fault system. HE: Hess escarpment. NPFB: North Panama fracture belt.

We conducted a field expedition across Central America from April to June 2021 to better understand and ecologically characterize the native populations of *A. aculeata* throughout the isthmus. We sampled leaves from natural populations across the region (Guatemala, Honduras, Nicaragua, Costa Rica and, Panama) for a total of 259 samples (**Supplementary Table S1**; **Figure 1**). The samples from the South American gene pool were obtained from previous studies and were included here solely for comparative purposes (**Suppementary Table S1**). A broader characterization of these samples, and of the South American *A. aculeata* gene pool in general, can be found in Díaz-Hernández et al. (2023) and Morales-Marroquín et al. (2025). The collections of these samples were registered in each country National Council of Biodiversity Patrimony. The leaves were dehydrated using silica gel and stored in paper bags at -20°C. Following Doyle and Doyle (1987), we extracted whole genomic DNA from 50 mg leaf samples. Agarose gel electrophoresis (1% w/v) with GelRed stain (Sigma-Aldrich) was used to assess DNA quality and integrity. We quantified and normalized DNA concentrations to 30 ng/μL using the dsDNA BR Assay quantification kit for the Qubit3 fluorometer (Invitrogen).

### GBS libraries and SNP discovery

Genomic libraries were prepared following the protocol of genotyping-by-sequencing using two restriction enzymes (ddGBS) as described by Poland et al (Poland et al., 2012). The combination of *MseI* and *PstI* (New England Biolabs) was used for the genomic library construction. The ddGBS library was quantified through RT-PCR on the CFX 384 Touch Real Time PCR (BioRad) equipment using a KAPA Library Quantification kit (KAPA Biosystems, cat. KK4824), and the fragments’ profiles were inspected using the Agilent DNA 1000 Kit on a 2100 Bioanalyzer (Agilent Technologies). The library was sequenced in an Illumina NextSeq2000 using the Illumina NextSeq1000/2000 kit, with single-end and 150 bp configurations.

The general sequencing quality and the presence of adapters were assessed with FASTQC (Andrews, 2010). The SNP discovery was performed following the *de novo* pipeline of the program Stacks v. 2.62 (Catchen et al., 2013) with similar filtering criteria. Due to the presence of adapters, sequences were trimmed to 90 bp. Trimming, quality control (removal of sequences with uncalled bases and with Phred scores <10), and demultiplexing were performed with the module *process_radtags*. For each sample, loci were assembled using the module *ustacks* considering minimum sequencing depth (-m) of 4, and distance between reads from the same loci (-N) of 5. A catalog of loci across samples was built with the module *cstacks*, considering the distance between locus (-n) of 4, and loci of the samples were compared to the catalog using *sstacks*. Candidate SNPs were identified using the module *populations* considering a minimum depth of 3, minor allele frequency of 0.01, the presence of SNP in at least 75% of the samples in each the sampling locations. To avoid explicit linkage only a single SNP was retained per GBS locus. Quality metrics (mean sequencing depth per locus and per sample, percentage of missing data per sample) were assessed with VCFTools (Danecek et al., 2011). Additionally, samples with more than 50% of missing genotypes were removed from the final data set, resulting in 259 samples for *A. aculeata*. We perform a data set imputation in TASSEL using LinkImpute (LD kNNi) imputation algorithm (Glaubitz et al., 2014).

### Populations genomic diversity and structure analyses

We used both putatively neutral and under-selection single nucleotide polymorphisms (SNPs) for the analysis of genomic diversity. The hierfstat package (Goudet, 2005) was employed in the R 4.1.0 platform (R Core Team, 2016) to calculate values for *H_0_* observed heterozygosity, *H_S_* gene diversity, *f* inbreeding coefficient, and their respective ranges, using 1000 bootstraps. Additionally, we utilized the adegenet 2.1.1 package (Jombart and Ahmed, 2011) and the poppr package (Kamvar et al., 2014) in the R 4.2.1 platform (R Core Team, 2016) to determine *A* allele number, *AR* allelic richness, *Ap* private alleles.

Population structure analyses were also conducted using both, neutral and under selection loci. The adegenet v. 2.1.6 package (Jombart, 2022) within the R 4.2.1 platform was utilized to perform the Discriminant Analysis of Principal Components – DAPC (Jombart and Ahmed, 2011), in addition to the methods mentioned earlier (R Core Team, 2016). Genetic groups based on Central America biogeographic zones and collection sites were considered during the DAPC analysis, and the optimal number of retained principal components was determined using the *α*-score. This technique allowed for the generation of scatter/density plots illustrating the distribution of populations. The hierfstat package (Goudet, 2005) was employed to compute Wright's F-statistics (Fixation index *F_ST_*, *F_IT_*, and *F_IS_*), and for the pairwise *F_ST_* values. Heatmaps illustrating the pairwise *F_ST_* results were generated using the heatmaply package (Galili et al., 2018) in R version 4.2.1 (R Core Team, 2016).

### Ecological niche modeling of *Acrocomia aculeata* in Central America

The distribution and occurrence data for *A. aculeata* was extracted from the following databases: GBIF (https://www.gbif.org/), the Missouri Botanical Garden (https://www.missouribotanicalgarden.org/)), and the New York Botanical Garden (https://www.nybg.org/), using the BIEN package (Maitner et al., 2018) for the R 4.1.0 platform (R Core Team, 2016).

After obtaining the data, we initially filtered the coordinates using the CoordinateCleaner package (Zizka et al., 2019) on the R platform version 4.1.0 (R Core Team, 2016). Subsequently, we added an occurrence point corresponding to each natural population site surveyed and observed in this study. Given *A. aculeata*'s extensive distribution across the continent, only coordinates from Central America were used. Finally, we employed a minimum of five occurrences for modeling, and spatial rarefaction was applied at twice the model cell resolution using the ENMTML package (Andrade et al., 2020) on the R 4.1.0 platform.

We employed the same 19 bioclimatic variables with a resolution of 2.5 arc minutes from the WorldClim database during the pre-processing stage of the models. To reduce collinearity between variables, Principal Component Analysis (PCA) was utilized, resulting in 19 principal components (PCs) and a correlation matrix. We selected 4 PCs that collectively explained at least 95% of the total variance, generating a new set of variables derived from PCA (Destro et al., 2020), also created using the ENMTML package. Subsequently, utilizing the "BUFFER" method within the ENMTML software, the model was fitted to a region defined by the polygon formed by the occurrence points.

In the ENMTML package, pseudo-absences were identified using the "env_const" method. These pseudo-absences are ecologically constrained to areas with reduced fitness values predicted by a Bioclim model (Booth et al., 2014). Thirty percent of the data was reserved for testing, while seventy percent was utilized for training to enhance model accuracy. Additionally, we employed a bootstrap approach, repeating the process ten times.

Several algorithms were employed during the processing step to predict the species' present suitability. Four algorithms were used to create the ENMs among these three groups: (1) Bioclim (Booth et al., 2014), which considered presence only; (2) Random Forest (Breiman, 2001); (3) Support Vector Machine (SVM) (Guo et al., 2005), which considered both presence and absence algorithms; and (4) MaxEnt (Phillips and Dudík, 2008), which considered presence and background algorithms. The models' quality was assessed using an analysis of the True Skill Statistics (TSS) (Allouche et al., 2006) and Area Under the Curve (AUC) (Metz, 1986) metrics.

Based on the suitability models, Ensembles (Araújo and New, 2006) were developed for the post-processing stage, we considered models with AUC > 0.75 and TSS > 0.5. This method can help with better planning and enables more robust decision making based on the responses from the models (Araújo and New, 2006). The average between the models was examined using the MEA technique, that is the ensemble with mean values of the models. To visualize the results of the ecological niche modeling, we used QGIS, an open-source geographic information system (QGIS Association, no date). QGIS allowed us to map the predicted suitability of *A. aculeata* across Central America with high spatial resolution. We also created a map using the Sentinel-2 10-meter Land Use/Land Cover rasters (Karra, 2021) from the European Space Agency (ESA), developed in collaboration with Esri Silver Partner Impact Observatory, to calculate the land use patterns across the Central American landscape and possible impacts in *Acrocomia* distribution. To infer land use and cover at the country level, we applied the Zonal Statistics tool in QGIS, which calculates pixel values from a raster layer within defined polygonal boundaries.

## Results

### SNP discovery and genomic diversity in *A. aculeata* in Central America

The sequencing of the ddGBS library for *A. aculeata* generated a total of 746,344,923 reads. After quality-control filtering, we identified 1523 SNPs. The mean depth per-sample was: 20.12× (SD ± 13.39×, 0.04% of missing data after imputation). Population genomic analyses of *A. aculeata* revealed clear patterns of genetic diversity and population structure across Central America, with the Central American gene pool showing distinct genomic differentiation from South American populations (**Supplementary Figure S1**; **Supplementary Figure S2**). Three major genomic groups were identified in the region: Mesoamerican, Costa Rican, and Panamanian (**Figure 1**; **Supplementary Figure S3**). Within these major clusters, subpopulations exhibited varying levels of genetic differentiation and diversity (**Table 1**). Central American *A. aculeata* populations exhibited moderate to low levels of heterozygosity compared to South American populations, possibly reflecting historical demographic events, genetic drift, and potentially limited gene flow due to regional conservation status and predatory practices in land use.

**Table 1:** Genetic diversity statistics of *Acrocomia aculeata* populations from 16 Central American locations. Parameters estimated from ddGBS data (259 individuals and 1523 SNPs). *H_0_* observed heterozygosity, *H_S_* gene diversity, *A* allele number, *AR* allelic richness, *Ap* private alleles, *f* inbreeding coefficient (Cockerham and Weir, 1993). The complete list of samples and their description can be found in **Supplementary Table S1**.

Major genomic groups		*n*	*H_0_*	*Hs*	*A*	*AR*	*Ap*	*f*
Mesoamerican		94	0.047	0.049	2154	1.205	2062	0.085
Costa Rican		70	0.044	0.040	1979	1.167	397	0.095
Panamanian		95	0.032	0.025	2026	1.155	607	0.089
Sub-regions								
Mesoamerican	Guatemalan North – GTN	12	0.042	0.033	1590	1.033	38	-0.131
	Guatemalan South – GTS	22	0.042	0.032	1747	1.032	109	-0.117
	Honduran West – HNW	5	0.047	0.039	1675	1.040	19	-0.162
	Honduran Center – HNC	19	0.047	0.042	1791	1.042	77	-0.001
	Honduran North – HNN	12	0.044	0.037	1734	1.037	68	-0.047
	Nicaraguan North – NCN	24	0.057	0.050	1809	1.050	71	-0.033
Costa Rican	Nicaraguan South – NCS	10	0.039	0.042	1721	1.042	41	0.212
	Costa Rican North West – CRNW	27	0.045	0.038	1814	1.038	99	0.027
	Nicoya Peninsula – CRNY	7	0.040	0.032	1670	1.033	18	-0.100
	Costa Rican North – CRN	13	0.050	0.041	1693	1.041	25	-0.134
	Costa Rican Center – CRC	10	0.042	0.033	1695	1.033	46	-0.128
	Costa Rica South – CRS	3	0.042	0.031	1618	1.032	6	-0.416
Panamanian	Osa Peninsula – CROS	48	0.028	0.020	1779	1.020	90	0.022
	Panamanian West – PNW	11	0.034	0.025	1607	1.026	19	-0.091
	Panamanian Center – PNC	23	0.032	0.023	1657	1.023	69	0.043
	Panamanian East – PNE	13	0.053	0.049	1685	1.049	101	-0.004

The Mesoamerican group included individuals from Guatemala to the Northwest region of Nicaragua, displayed the highest overall genetic diversity. Regional subpopulations within this group exhibited subtle genetic differences. For instance, individuals from southern Guatemala displayed slightly lower observed heterozygosity (*H_0_*) compared to other Mesoamerican subpopulations, indicating potential genetic isolation or drift in this region due to land use and landscape shift. In contrast, individuals from northern Nicaragua (NCN) exhibited relatively high levels of genetic diversity, as evidenced by higher *H_0_*, lower *f* coefficient, and allelic richness (*AR*), suggesting historical connectivity or admixture. Furthermore, southern Guatemala (GTS) have more private alleles than all other populations in Central America. The highest value of private alleles (*Ap*) within the major genomic groups was from the Mesoamerican (*Ap* = 2062) suggesting unique genetic variation within this population and more genomic diversity. The fixation index (*f* = 0.086), indicated moderate genetic differentiation by inbreeding.

The Costa Rican group including populations from Nicaraguan South (NCE) to the Central Valley and southern Costa Rica (CRS) showed intermediate levels of genetic diversity and differentiation compared to the other groups. Inside the Costa Rican group, the highest and lowest diversity was observed in the sub-regions Costa Rican north (CRN) and Nicaraguan South (NCS), respectively. Genetic substructure was evident, with individuals from the Nicoya Peninsula (CRNY) showing reduced gene flow and possible isolation. The Costa Rican group has the lowest number of private alleles (*Ap* = 397). The fixation index (*f* = 0.095) was slightly elevated indicating genetic differentiation compared to the Mesoamerican and the Panamanian groups. These results are consistent with previous SSR-based studies in the region (Navarro-Cascante et al., 2023).

The Panamanian group, spanning from southern Costa Rica (Osa Peninsula) to eastern Panama, exhibited lower levels of genetic diversity (*H_0_* and *H_S_*) in the isthmus. The Osa Peninsula (CROS) exhibited the lowest heterozygosity (*H_0_* = 0.028) and allelic richness (*AR* = 1.020). Interestingly, eastern Panama (PNE) individuals showed relatively higher genetic diversity (*H_0_* = 0.053, *AR* = 1.049), possibly reflecting historical introgression from other populations or ongoing migration dynamics.

### Population structure of *A. aculeata* in the Central American isthmus

The Discriminant Analysis of Principal Components (DAPC) revealed three well-defined genomic clusters (**Figure 1B**; **Supplementary Figure S3**). Individuals from Guatemala and Honduras grouped primarily within the Mesoamerican cluster (orange), while those from Costa Rica formed a distinct cluster (blue). Notably, Nicaragua, particularly the northern populations, displayed a high degree of admixture between the Mesoamerican and Costa Rican clusters, indicating a transition or hybrid zone where gene flow between these groups is ongoing or occurred in the recent past. In contrast, the Panamanian populations (gray) appeared clearly differentiated, with minimal admixture from neighboring regions (**Figure 1**; **Supplementary Figure S3**). This genetic distinctiveness suggests restricted gene flow and possible historical isolation, likely shaped by ecological or geographic barriers near the southern limit of the isthmus.

These results indicate a pattern of genetic continuity by gene flow between the Mesoamerican and Costa Rican groups, interrupted by a stronger genomic divergence toward Panama, supporting the presence of both connectivity and isolation dynamics along the Central American isthmus. It is particularly interesting that the Osa Peninsula, located in the Pacific region of Costa Rica (CROS population; **Figure 1**), harbors *A. aculeata* individuals with a distinct genetic identity compared to other Costa Rican populations. The genotyped palms from this peninsula cluster more closely with the Panamanian genetic group rather than with the Costa Rican cluster, suggesting a demographic history linked with the Panamanian group. This pattern may reflect a founder effect, in which a few colonizing individuals from Panama established the Osa Peninsula population, carrying only a subset of the genetic diversity from the source population.

**Figure 2:**
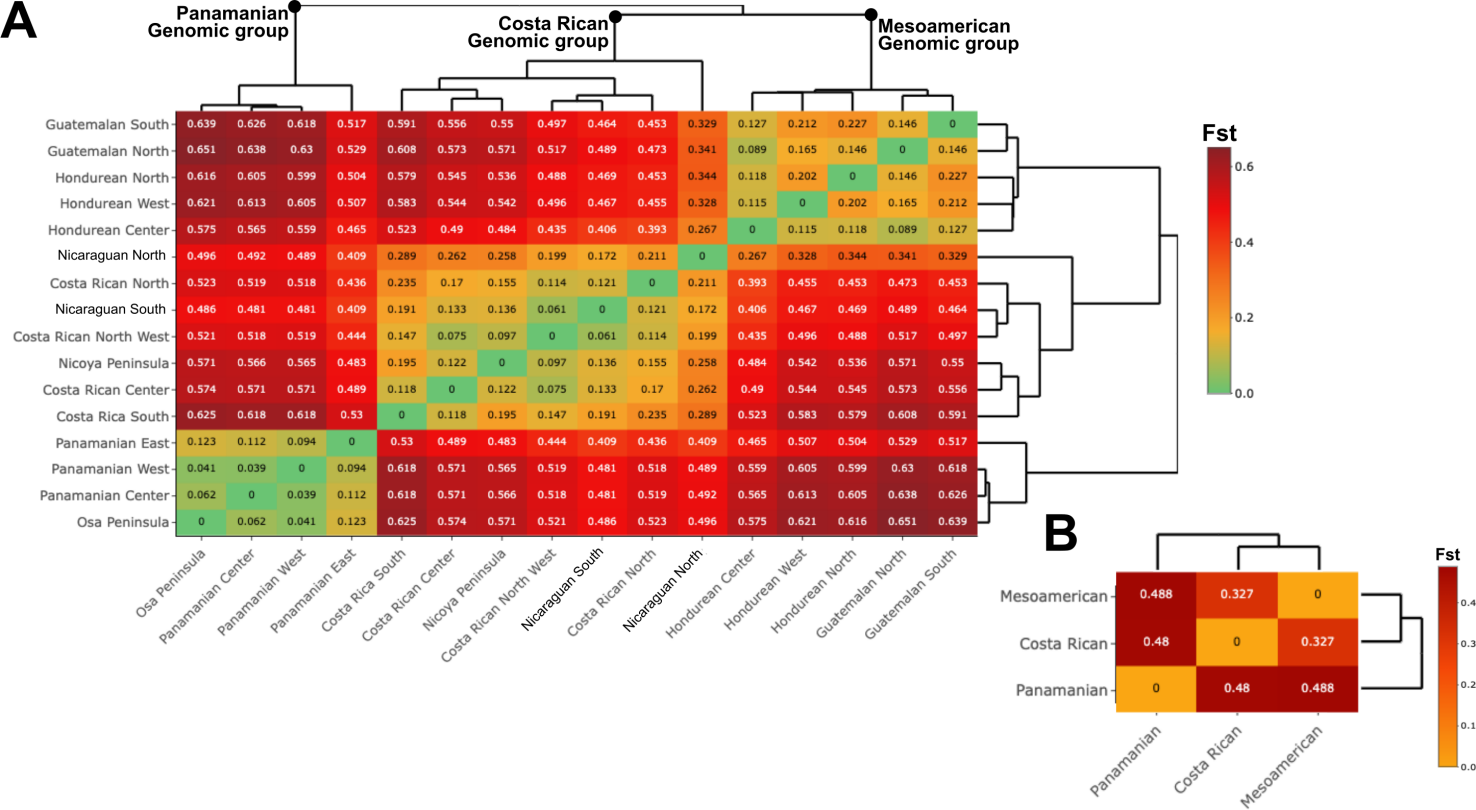
Dendrogram and heatmap based on fixation index values *F_ST_* comparing *A aculeata* sampling locations. **(A)***F_ST_* was calculated using the whole set of markers (1523 SNP) shows separation between the Mesoamerican, Costa Rican and, Panamanian subpopulations. **(B)***F_ST_* shows separation between the Mesoamerican, Costa Rican and, Panamanian groups.

Pairwise *F_S_*_T_ values also revealed clear genetic differentiation among *A. aculeata* populations across Central America, supporting the presence of three major genomic groups: Mesoamerican, Costa Rican, and Panamanian (**Figure 2**). The Mesoamerican cluster includes populations from Guatemala and Honduras, which show relatively low *F_ST_* values among themselves (0.18–0.25), indicating higher genetic connectivity and shared ancestry. Populations from Costa Rica form an intermediate cluster, showing moderate *F_ST_* values (0.20–0.30) with northern populations from Nicaragua, suggesting that Nicaragua, especially its northern region, acts as a genetic transition zone between the Mesoamerican and Costa Rican genomic groups. This pattern is consistent with the admixture signals detected in the DAPC analysis. In contrast, the Panamanian populations exhibit the highest *F_ST_* values (>0.45) relative to both Costa Rican and Mesoamerican clusters, supporting the strong genetic differentiation between the three genomic groups and limited gene flow with northern populations.

Despite their geographic proximity to southern Costa Rica (less than 50 km), the Panamanian populations remain genetically distinct. The Panamanian genomic group exhibited lower levels of genetic differentiation among themselves (0.03-0.06) compared to those from the Mesoamerican and Costa Rican groups. These low *F_ST_* values indicate higher genetic connectivity and suggest that gene flow is more extensive. Such a pattern reflects the continuous distribution of *A. aculeata* across Panama and the presence of historical genetic maintenance and exchange among sites. This relative genetic homogeneity within Panama contrasts with the stronger structure observed in the northern regions.

The Nicaraguan Depression and the Talamanca Cordillera act as biogeographic barriers shaping the genetic structure of *A. aculeata* in Central America (**Figure 1**). The Nicaraguan Depression, a lowland dry corridor of volcanic plains, limits gene flow between Mesoamerican and Costa Rican populations, as reflected by moderate *F_ST_* values and evidence of admixture in northern Nicaragua. In contrast, the Talamanca Cordillera, a high-elevation range exceeding 4,000 masl, very humid, creates a genetic discontinuity between Costa Rican and Panamanian groups. Our results reveal a north–south gradient of genetic divergence, with gradual connectivity between Mesoamerica and Costa Rica, followed by sharp differentiation toward Panama, reflecting historical isolation across the Central American landscape.

### Environmental suitability based on ecological niche modeling of *A. aculeata* in Central America

**Figure 3:**
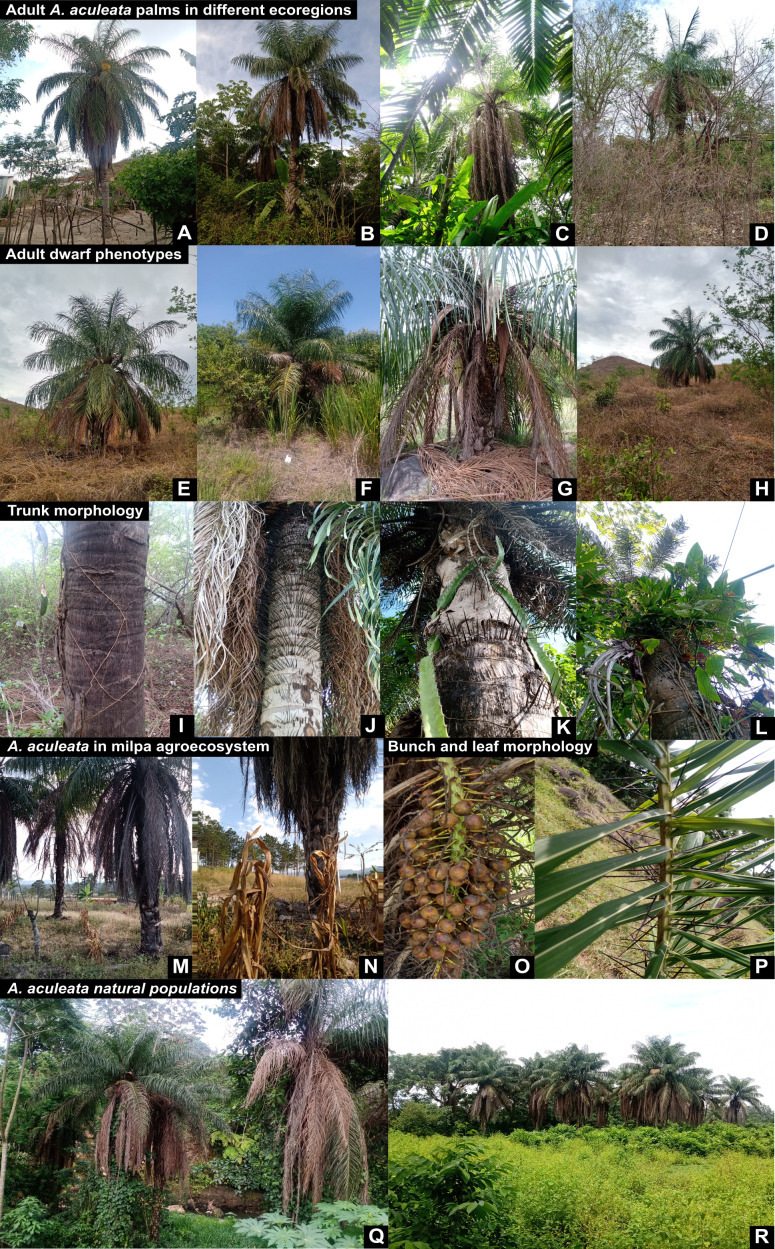
Characteristics of adult plants of *Acrocomia aculeata* in Central America. **(A–D)**: Adult palms in different ecoregions. **(E–H)**: Adult dwarf phenotypes. **(I–L)**: Trunk morphology (stipe). **(M–N)**: *A. aculeata* in milpa agroecosystems. **(O–P)**: Bunch and leaf morphology. **(Q–R)**: *A. aculeata* natural populations.

Central American populations of *A. aculeata* are primarily found in transitional zones between dense forest cover and rangelands or croplands, demonstrating their adaptability to human-modified landscapes across diverse ecoregions. Our sampled populations ranged from 10 to 1,400 meters above sea level (masl) and were mainly associated with moist and dry broadleaf forests, as well as montane coniferous ecosystems (**Figure 3**). In lowland moist forests (0–300 masl), we also encountered other canopy neotropical palm genera such as *Attalea*, *Bactris* and *Syagrus*, although these tended to occur in areas with denser forest cover, while *Acrocomia* was more commonly found on forest edges or in more open and modified landscapes. Compared to the typical phenotypes of South American populations, Central American *A. aculeata* individuals, often display a spiny trunk (stipe) that has less, or even lacks, of the persistent leaf sheath fragments seen in southern populations. Additionally, some Mesoamerican populations, particularly in Guatemala, Honduras and, Nicaragua exhibit adult dwarf phenotypes, with individuals reaching less than two meters in height (**Figure 3**). The most abundant populations were observed in Costa Rica and Panama.

**Figure 4:**
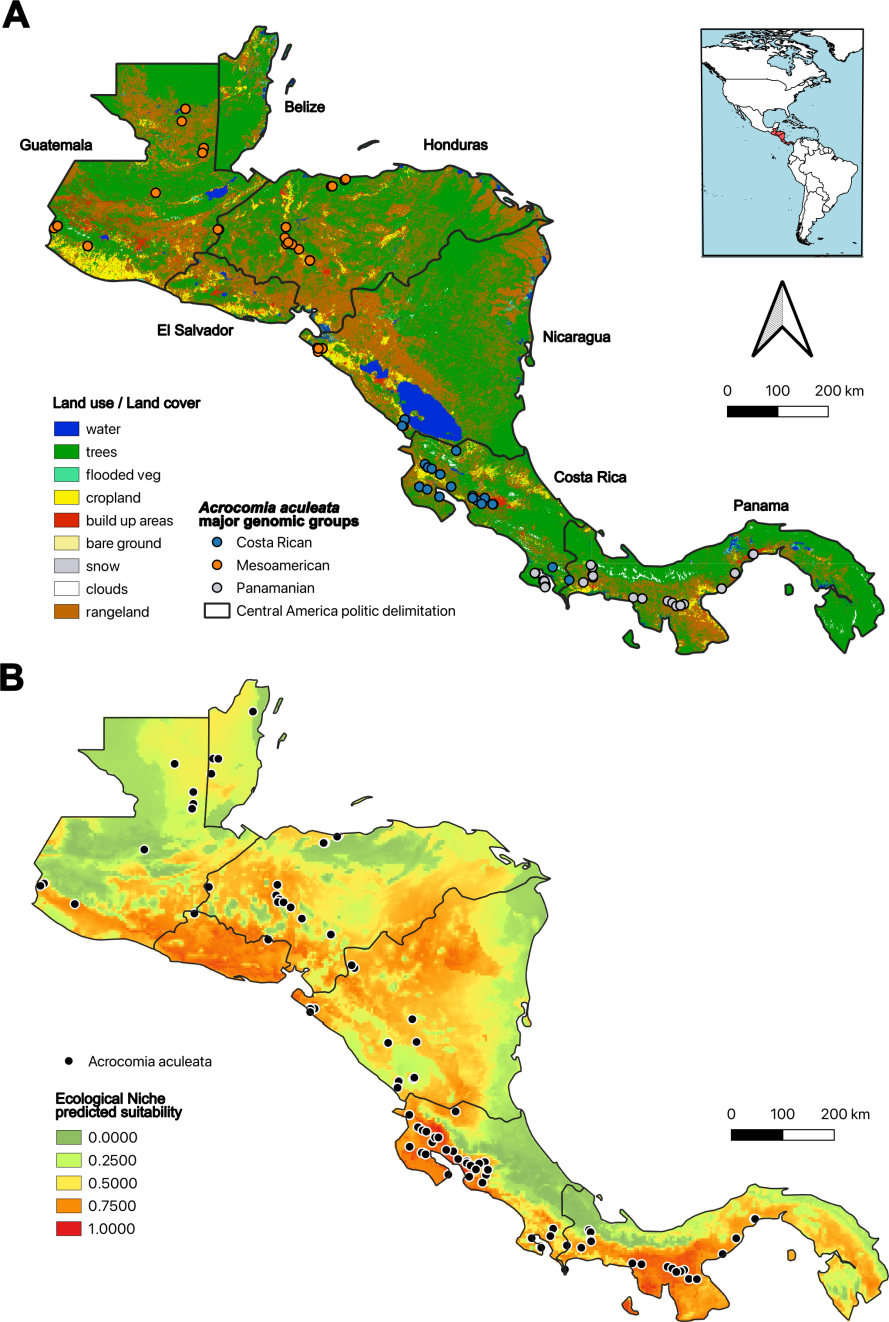
Land-used map and ecologic niche modeling (ENM) of *Acrocomia aculeata* in Central America. **(A)** Land use of Central America using Sentinel-2 10m Land Use/Land Cover Time Series (Karra, 2021). **(B)** Ecologic niche model using the MEA method, ensemble with mean values of the models (TSS: 0.5–0.8; AUC: 0.8–0.9). Sampling locations (points on the map) include field collections from this study as well as occurrence records obtained from the Global Biodiversity Information Facility (GBIF; https://www.gbif.org/), the Missouri Botanical Garden (https://www.missouribotanicalgarden.org/), and the New York Botanical Garden (https://www.nybg.org/), accessed through the BIEN package (Maitner et al., 2018).

Ecological niche modeling identified that *A. aculeata* is predominantly adapted to the Central American lowlands, particularly the Pacific region, where climatic conditions are characterized by pronounced seasonality in precipitation (**Figure 4**). The final ensemble model, selected using the MEA technique, ensemble with mean values of the models, due to its superior performance (TSS: 0.5–0.8; AUC: 0.8–0.9), highlights these areas as potential ecological corridors and proper areas for its cultivation (**Supplementary Table S2**). The model for *A. aculeata* showed strong overlap with the Köppen climate classification Aw (tropical savanna), indicating that the species is primarily distributed in warm, seasonally dry regions characterized by pronounced wet and dry periods (Beck et al., 2023). This pattern highlights the species’ preference for areas with high solar radiation and moderate annual precipitation, typically ranging between 1000–2000 mm, conditions that favor open-canopy environments where *A. aculeata* thrives.

The spatial overlap between highly suitable zones and the distribution of modified landscapes by human use suggests that there is a close relationship of *A. aculeata* with human settlements (**Figure 4B**). Also the abundancy of populations of Panama and Costa Rica maybe related to tree cover, with 67.5% and 61.9% respectively. In contrast, countries like El Salvador and Guatemala, with higher proportions of croplands (12.6% and 7.5%, respectively) and built areas (11.4% and 6.4%, respectively), display fragmented landscapes that may restrict dispersal and promote genetic differentiation (**Table 2**). It is important to note that Guatemala, El Salvador, Honduras, and Nicaragua have the lowest tree cover, the highest levels of habitat fragmentation and urbanization in the Isthmus, and relatively weak environmental policies. Rangelands, which dominate in Nicaragua (33.2%) and Honduras (35.6%), could function as intermediate habitats maintaining partial connectivity, although their effectiveness likely depends on the intensity of anthropogenic pressure and land conversion (**Table 2**). These patterns broadly align with niche model predictions, indicating that regions with more continuous natural vegetation are also those with higher genetic cohesion. Together, the patterns of genetic structure, admixture, and environmental suitability reveal a dynamic biogeographic history for *A. aculeata* in Central America, shaped by both biogeographic barriers and contemporary landscape configurations.

**Table 2:** Land cover classification across Central America based on Sentinel-2 10-meter Land Use/Land Cover rasters (Karra, 2021).

Classes	Guatemala		Belize		El Salvador		Honduras		Nicaragua		Costa Rica		Panama	
	Km^2^	%	Km^2^	%	Km^2^	%	Km^2^	%	Km^2^	%	Km^2^	%	Km^2^	%
Water	1772.7	1.5	911.5	3.9	538.0	2.5	2223.7	1.9	11108.9	8.4	972.1	1.9	2321.2	3.1
Trees	63530.1	54.5	16495.4	70.9	7725.2	35.5	64907.2	55.6	70504.1	53.5	32425.2	61.9	50459.8	67.5
Flooded Veg	303.4	0.3	611.8	2.6	63.2	0.3	376.6	0.3	804.4	0.6	49.8	0.1	416.5	0.6
Crops	8764.4	7.5	845.0	3.6	2732.8	12.6	3689.0	3.2	3879.5	2.9	3404.0	6.5	2262.3	3.0
Built Area	7487.4	6.4	338.9	1.5	2469.1	11.4	3813.9	3.3	1494.7	1.1	2222.1	4.2	1705.0	2.3
Bare ground	41.7	0.0	4.7	0.0	7.0	0.0	33.9	0.0	56.2	0.0	24.6	0.0	48.9	0.1
Snow Ice	0.0	0.0	0.0	0.0	0.0	0.0	0.0	0.0	0.0	0.0	0.0	0.0	0.0	0.0
Clouds	214.0	0.2	2.2	0.0	0.3	0.0	129.3	0.1	302.5	0.2	744.7	1.4	1035.2	1.4
Rangeland	34478.3	29.6	4060.5	17.4	8205.6	37.7	41469.5	35.6	43723.1	33.2	12508.4	23.9	16469.1	22.0
	116591.9	100.0	23270.0	100.0	21741.3	100.0	116643.1	100.0	131873.3	100.0	52350.7	100.0	74718.1	100.0

## Discussion

The geological patterns and processes of Central America has profoundly influenced the biogeographic history of Neotropical flora, while population genomics seeks to uncover the microevolutionary processes that structure, connect, and shape populations through shared evolutionary histories. Central America, a globally recognized biodiversity hotspot, offers a unique context to explore these dynamics. In this study, we used the Neotropical palm *A. aculeata* as a model to investigate how the genetic diversity of a widely distributed lowland plant species is organized across the isthmus. This work presents the first comprehensive genomic survey of an open-canopy, lowland plant species across the entire Central American corridor, employing SNP markers to evaluate genome-wide diversity and ecological niche modelling. In the following sections, we discuss our key findings on the population structure of *A. aculeata*, emphasizing their evolutionary implications and potential applications for conservation and breeding strategies.

### Biogeographic patterns and colonization history of *A. aculeata* in the Isthmus

*Acrocomia aculeata* inhabit tropical lowlands (0–1300 masl) across Central America. Their habitats shift from moist broadleaf forests to semi-arid environments. Population genomic analyses revealed that the Central American gene pool represents a genetically differentiated lineage from South American populations, likely shaped by historical isolation and limited contemporary gene flow. The Central American gene pool is composed of three major genomic groups and notably, the Nicaraguan Depression and the Talamanca Cordillera (Costa Rica) appear to act as major biogeographic barriers influencing the genetic structure of *A. aculeata* in the region (**Figure 1**). Central American populations of *A. aculeata* shows lower genetic diversity than South American ones, a pattern consistent with its biogeographic history. South America, particularly the Brazilian Shield, is considered the center of origin and long-term diversification for the genus, where large, continuous populations have persisted over evolutionary timescales, maintaining high levels of allelic richness and genetic variation (Díaz et al., 2021; Monge-Castro et al., 2025). Similar patterns in genetic structure and phylogenomic studies are also present in other palms genera such as *Chamaedorea*, *Geonoma* (Cano et al., 2022) and other clade species like entomofauna (Cano et al., 2018; Beza-Beza et al., 2021), arthropods (Crews and Esposito, 2020), Squamata (Saldarriaga-Córdoba et al., 2017), Anura (Wang et al., 2008; Mendoza-Henao et al., 2020), ichtyofauna (Matamoros et al., 2015), and parasites (Yisrael et al., 2024).

The origins and diversification of *Acrocomia* remain uncertain in the Isthmus, though current evidence supports a South American origin, particularly in the tropical lowlands of the Brazilian Cerrado biome (dry broadleaf forests, savannas, and xeric shrublands), 23 million BP ago, where species richness, genetic diversity is higher compared to Central American populations. These patterns suggest a south-to-north radiation across the Neotropics, eventually reaching Central America (Morcote-Ríos and Bernal, 2001), a dispersal dynamic also observed in other Bactridinae palms and tropical plant lineages (Gentry, 1982; Eiserhardt et al., 2011; Pérez-Escobar et al., 2019; Cano et al., 2022).

Our results revealed greater genetic diversity, higher number of private alleles, and higher structuration within the Mesoamerican genomic group compared to the Panamanian group, suggesting an early and complex colonization history. Lower Central America (Costa Rica and Panama) began forming over 100 million years ago from oceanic and volcanic terranes that progressively accreted along the western Caribbean plate. By the Eocene (50–38 million BP), emergent land formed an island archipelago separated from both continents by wide marine corridors (Marshall, 2007; Alvarado and Cárdenes, 2016). These islands served as early stepping stones for overwater plant dispersal, including ancestral *Acrocomia* lineages moving northward from South America (Bagley and Johnson, 2014). Between the Miocene and Early Pliocene (24–5 million BP), periods of land emergence led to the formation of most landmasses that now comprise the Central American Isthmus. By the Late Miocene (~9 Mya), initial terrestrial connections between North and South America had been established (Bagley and Johnson, 2014; Bacon et al., 2015).

These geologic events have driven similar patterns of genetic differentiation across various taxa within each tectonic block. Northern Central American flora is characterized by strong genetic structure and divergence, with evidence of numerous refugia and the emergence of cryptic species. Mid-Central America exhibits high levels of population differentiation associated with intense volcanic activity, while the Panamanian region reflects patterns of North–South migration, contributing to high species richness and active speciation processes (Gutiérrez-García and Vázquez-Domínguez, 2013). This land emergence facilitated terrestrial migrations and genetic exchange, marking a key period for *Acrocomia*’s establishment in Central America. The Mayan block, which already harbored rich tropical vegetation, likely acted as a northern dispersal cradle for these north-to-south expansions of *Acrocomia* to eventually new territories in Lower Central America (Chorotega anc Chocó Block) (Stebbins, 1974; Bagley and Johnson, 2014).

Later, with the uplift of the Chocó block and the final closure of the Isthmus of Panama (~5 million BP), migration routes reversed direction (**Figure 1C**), enabling southward gene flow and recontact between previously isolated lineages (Bagley and Johnson, 2014; Montes et al., 2015; Alvarado and Cárdenes, 2016). Together, these tectonic and climatic processes generated a mosaic of dispersal corridors and barriers that shaped the contemporary genetic structure of *A. aculeata*. The northward migration during Miocene land emergence and the later southward exchange during the Great American Biotic Interchange (GABI) produced the distinct genomic lineages now observed across the Central American isthmus. Based on the floristic character of the region, Gentry (1982) postulated more than forty years ago that South America is an important source of lineages in Central America (Pérez-Escobar et al., 2019), a pattern also observed for *Acrocomia*. This is also evident in the contrasting *F_ST_*, private allele distribution, and genomic structure of the three mayor genomic groups identified in our study.

The strong genetic differentiation observed among the three major genomic groups, particularly between the Meso/Costa Rican and Panamanian clusters (mean *F_ST_* > 0.4), suggests limited contemporary gene flow across the isthmus. This pattern is consistent with the species’ present reproductive ecology, *A. aculeata* is predominantly allogamous, relying on weevil mediated pollination and cattle, human, and gravity-driven seed dispersal (Lentz, 1990; Göldel et al., 2016; Carreño-Barrera et al., 2025). These mechanisms promote local gene exchange but restrict long-distance dispersal, especially across fragmented or topographically complex landscapes such as Central America. The combination of localized pollen flow and limited seed movement likely reinforces the observed genomic structure and may contribute to incipient reproductive isolation between regional lineages. However, admixture patterns in transition zones, such as northern Nicaragua, indicate that gene flow can still occur where populations remain connected. There is currently no evidence of ploidy differences or hybrid infertility among groups, suggesting that isolation is primarily geographic and ecological rather than genetic or cytological.

Central American populations likely originated from northward dispersal events from South America, involving founder effects and sequential colonization across emerging landmasses of the Central American Isthmus. Our results reinforce the importance of South America as a historical source of neotropical lineages and highlight how land emergence and geographic barriers have contributed to genetic differentiation within *A. aculeata*. These findings have important implications for conservation and breeding, underscoring the need to maintain habitat connectivity and preserve genetic resources to support the resilience and adaptive potential of populations facing rapid environmental and anthropogenic change.

### Landscape dynamics and niche suitability of *A. aculeata*

Understanding the present spatial distribution of *A. aculeata* across Central America requires the integration of genomic data with landscape dynamics and ecological niche modeling. While climate variables define the species' fundamental niche, land use and habitat transformation modulate its realized niche, shaping patterns of connectivity, gene flow, and potential local adaptation (Rodriguez-Cabal et al., 2012). *A. aculeata* is suited to the Central American lowlands, where climatic conditions are characterized by high temperatures and marked seasonality in precipitation. This climate regime favors individuals with drought tolerance and physiological flexibility, traits that align with *A. aculeata*’s persistence in open, sun-exposed habitats such as rangelands, croplands, and transitional forest zones. Selective signatures for these traits have already been described for the species, showing associations with bioclimatic variables such as annual temperature (BIO1), diurnal temperature range (BIO2), annual precipitation (BIO12), and particularly precipitation in the driest month (BIO14) (Morales-Marroquín et al., 2025). The observed genetic structuring among Central American populations may be explained by the fragmented landscapes across the isthmus, which likely reinforced population differentiation and contributed to the high *F_ST_* values and elevated inbreeding coefficients.

The widespread distribution and ecological plasticity observed in *A. aculeata* likely reflect both its historical dispersal pathways and its capacity to persist under altered selective pressures in human-modified landscapes. Although phylogenetic biome conservatism is common among tropical palms, where species typically diversify within rather than across biomes (Cássia-Silva et al., 2019), *A. aculeata* appears to be an exception, likely due to recent human interactions. Historical long-distance seed dispersal by megafauna and contemporary human and cattle-mediated dispersal may have contributed to the species' expansive range and ecological plasticity (Costa et al., 2025). Our model reveal that *A. aculeata* maintains abundant populations in regions dominated by continuous forest cover, such as Panama and Costa Rica, where landscape integrity likely promotes greater genetic connectivity. In contrast, countries with extensive habitat fragmentation, such as Guatemala, El Salvador, Honduras, and Nicaragua, exhibit higher levels of genetic differentiation, likely resulting from disrupted dispersal pathways and potential population bottlenecks.

The ecological resilience of *A. aculeata* is further evidenced by its frequent occurrence in rangelands, croplands in different Central American ecoregions and particularly within home gardens (**Figure 3A**) and milpa agroecosystems (**Figure 3M, N**). Milpa is an indigenous Mesoamerican polycultures that combine maize, beans, and squash in cyclical cultivation regimes that promote sustainable land use (**Figure 3A, M, N**) (Grof-Tisza et al., 2024). The occurrence of *A. aculeata* in human-managed landscapes suggests a long-standing interaction with traditional agricultural systems, potentially indicating early stages of incipient domestication in some populations. Its cultivation in home gardens further underscores their importance in preserving high levels of inter- and intraspecific plant genetic diversity, particularly through the conservation of traditional crop varieties and landraces (Galluzzi et al., 2010). Furthermore, the traditional indigenous practice of felling adult palms to harvest sap for fermentation, still widespread in Central America and particularly intense in Costa Rica, has added further pressure on the species management (Balick, 1990; Navarro-Cascante et al., 2023).

This co-occurrence and management emphasizes the role of indigenous agroecosystems in maintaining and modifying agrobiodiversity and facilitating gene flow in culturally significant plant species across the region. These evolutionary and ecological patterns are also seen in several edible plant lineages domesticated in Mesoamerica, such as maize, beans, cucurbits, and chili (*Capsicum* spp.), where biogeographic history and human selection jointly shaped their genetic and phenotypic diversity across tropical landscapes (Araceli et al., 2009; Blair et al., 2009; Bedoya et al., 2017; Carrizo García et al., 2022; Castellanos-Morales et al., 2024; Domic et al., 2024).

The actual expansion of the agricultural frontier in Central America, driven largely by industrial crops such as sugarcane and African oil palm, has profoundly altered the Pacific coast and lowlands landscape (**Figure 4B**) and likely contributed to the fragmentation and demographic decline of *A. aculeata*, and other native costal species (Pirker et al., 2016; Castellanos-Navarrete et al., 2019, Castellanos-Navarrete et al., 2021). These land-use changes have replaced diverse forest mosaics and secondary habitats with extensive monocultures, thereby restricting the potential distribution of the species and interrupting natural dispersal routes. Our findings of low population densities and strong genetic differentiation across fragmented landscapes in countries such as Guatemala, Honduras and Nicaragua reflect these patterns of habitat loss and isolation. In contrast, the relatively higher population richness found in protected areas of Costa Rica highlights the importance of landscape integrity promoted by strong environmental policies for maintaining gene flow. These results suggest that the proliferation of large-scale agriculture has not only limited the species’ ecological niche but has also disrupted its evolutionary potential by reducing connectivity and effective population sizes. Given that, *A. aculeata* tends to persist in potentially disturbed habitats but at low densities, the cumulative impact of agricultural expansion likely exacerbates genetic erosion across much of its Central American range.

By linking spatially explicit genomic patterns with high-resolution land cover data and niche suitability projections, we show that biogeographic barriers, environmental variables and anthropogenic pressures jointly shape *A. aculeata*’s distribution. Niche modeling predicts highest habitat suitability in the Pacific lowlands of Central America, regions that align with the core areas of genomic diversity identified in our study. These overlapping zones underscore the potential of ecological modeling to guide conservation priorities and agroforestry planning. From a conservation and breeding perspective, our findings highlight the need for localized management strategies, as the Central American gene pool exhibits unique genomic and phenotypic characteristics. Given the strong genetic structure observed among the major regional groups, particularly the high differentiation of the Panamanian group, seed transfer between regions should be avoided to prevent genetic homogenization and preserve locally adapted lineages. In regions with high fragmentation, such as Guatemala, Honduras, and Nicaragua, reforestation efforts and stronger environmental policies should prioritize the restoration of habitat corridors to enhance gene flow and preserve genetic diversity. In contrast, Panama and Costa Rica, with stronger and more comprehensive environmental frameworks, exemplify how conservation strategies focused on maintaining habitat integrity and anticipating climate-driven shifts in suitable niches can support biodiversity conservation (Morales-Marroquín et al., 2022).

This study presents a comprehensive analysis of the population genomics and biogeographic history of *A. aculeata* in Central America, revealing how geological processes, environmental heterogeneity, and contemporary land use have shaped the species’ genetic structure and ecological distribution. By integrating SNP-based genomic data with ecological niche modeling and land use classification, we uncover both conserved and divergent population patterns, highlighting the roles of geographic isolation, environmental gradients, and anthropogenic pressures in driving gene flow and local adaptation. Ultimately, this research provides a foundation for more informed genetic resource management of *A. aculeata* in Central America and positions the species as a valuable Neotropical model for understanding how plant species respond to landscape modification in a neglected tropical biodiversity hotspot.

## Data Availability

The data presented in this study are publicly available in the NCBI BioProject repository under accession number PRJNA1255231. The Excel spreadsheet containing the sample list with geographical locations and characteristics is available in the public repository: https://doi.org/10.6084/m9.figshare.28886687.
