# Supplementary material for: Genomic structure, ecological suitability and biogeographic history of the palm Acrocomia aculeata across Central America
Source: Front Plant Sci. 2026 Jan 27;16:1724384. doi: 10.3389/fpls.2025.1724384 (PMC12886420; doi:10.3389/fpls.2025.1724384)

Supplemental Material

Genomic structure, ecological suitability and biogeographic history of the palm *Acrocomia aculeata* across Central America

Jonathan A. Morales-Marroquín, Erick Rene Lopez de Paz, Rocio Silva-Rivera, Ana Flávia Francisconi, Roger Alejandro Orellana-Hernandez, José M. Palacios, Emmanuel Araya-Valverde, Elizabeth Arnáez-Serrano, João Victor da Silva Rabelo-Araujo, Caroline Bertocco Garcia, Matheus Scaketti, Carlos A. Colombo, Brenda Gabriela Díaz-Hernández, José Baldin Pinheiro, Maria Imaculada Zucchi

Supplementary Table S1. *Acrocomia* species samples. Geographical location and biome of the *Acrocomia aculeata* samples in Central America. (Excel spreadsheet on public repository https://doi.org/10.6084/m9.figshare.28886687)

Supplementary Table S2. Evaluation table with the algorithms and ensembles applied in the Ecological Niche Modeling of Acrocomia aculeata in Central America. BIO = Bioclim; MEA = ensemble with mean values of the models; MXS = MaxEnt; RDF = Random Forests; SVM = Support Vector Machine. AUC = Area Under the Curve, and TSS = True Skill Statistics.

Sp	Algorithm	Threshold	Partition	AUC	TSS	AUC_SD	TSS_SD
Acrocomia_aculeata	**BIO**	**MAX_TSS**	**BOOT**	**0.943103**	**0.886207**	**0.03729**	**0.07458**
Acrocomia_aculeata	**MEA**	**MAX_TSS**	**BOOT**	**0.921998**	**0.717241**	**0.027655**	**0.045399**
Acrocomia_aculeata	**MXS**	**MAX_TSS**	**BOOT**	**0.802021**	**0.551724**	**0.037098**	**0.082886**
Acrocomia_aculeata	**RDF**	**MAX_TSS**	**BOOT**	**0.943876**	**0.758621**	**0.0302**	**0.072696**
Acrocomia_aculeata	**SVM**	**MAX_TSS**	**BOOT**	**0.882283**	**0.668966**	**0.017268**	**0.033314**

Supplementary Figure S1. Discriminant analysis of principal components (DAPC) for *A. aculeata* populations of Central America and South America showing the separation between major gene pools. **Populations acronyms: Central American Group** = GT: Guatemalan South, G: Guatemalan North, HN: Honduras, NC: Nicaragua, CR: Costa Rica, CC: Costa Rica Osa Peninsula, PN: Panama. **South American Group** = AM: *Acrocomia aculeata* Amazonas, ACINTU: *Acrocomia intumescens* Paraíba, AT: *Acrocomia totai* São Paulo, EL: *Acrocomia totai* Mato Grosso do Sul, EC: *Acrocomia aculeata* Minas Gerais, RR: *Acrocomia aculeata* Roraima.

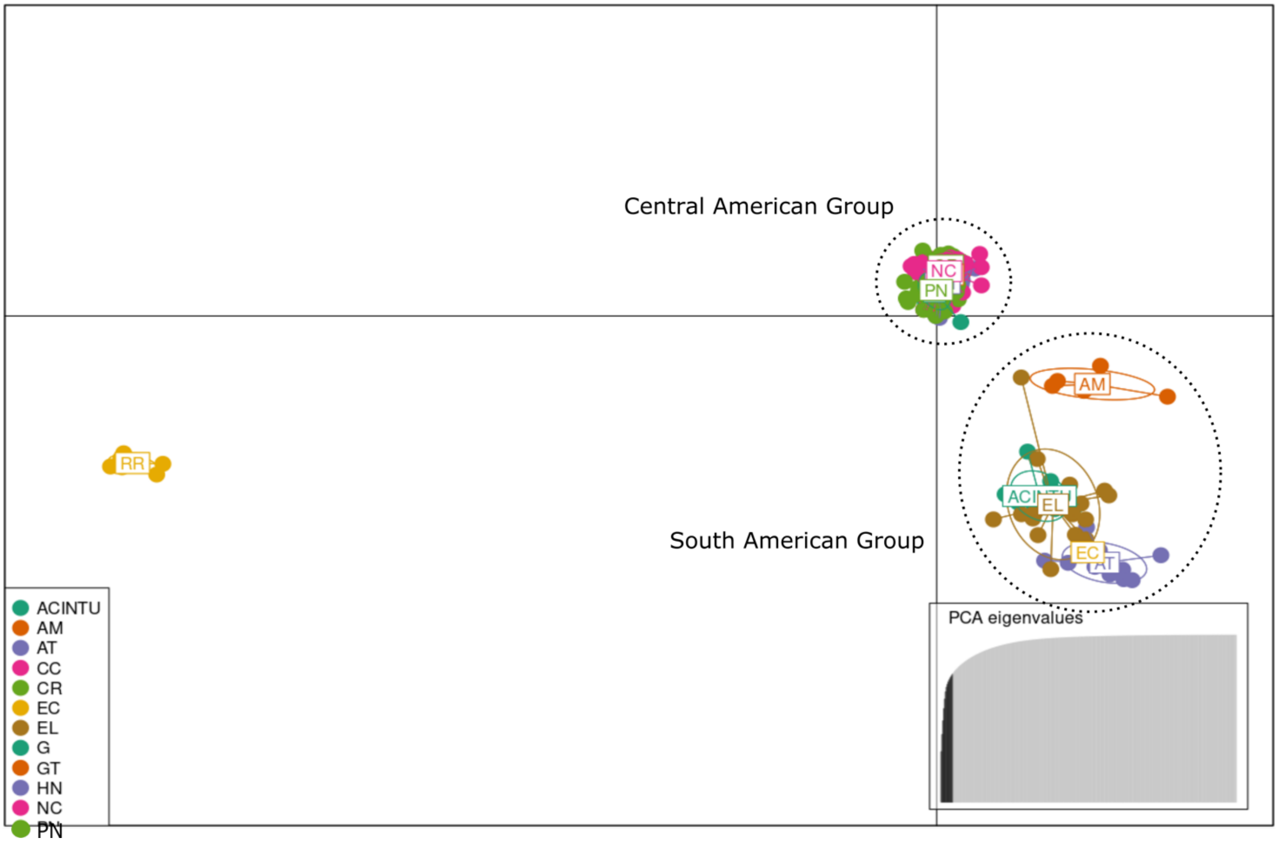

Supplementary Figure S2. Dendrogram and heatmap based on fixation index values F_{ST} comparing *A. aculeata* sampling locations in Central America with South American populations. **Populations acronyms: Central American Group** = GT: Guatemalan South, G: Guatemalan North, HN: Honduras, NC: Nicaragua, CR: Costa Rica, CC: Costa Rica Osa Peninsula, PN: Panama. **South American Group** = AM: *Acrocomia aculeata* Amazonas, ACINTU: *Acrocomia intumescens* Paraíba, AT: *Acrocomia totai* Mato Grosso do Sul, EL: *Acrocomia aculeata* São Paulo, EC: *Acrocomia aculeata* Minas Gerais, RR: *Acrocomia aculeata* Roraima.

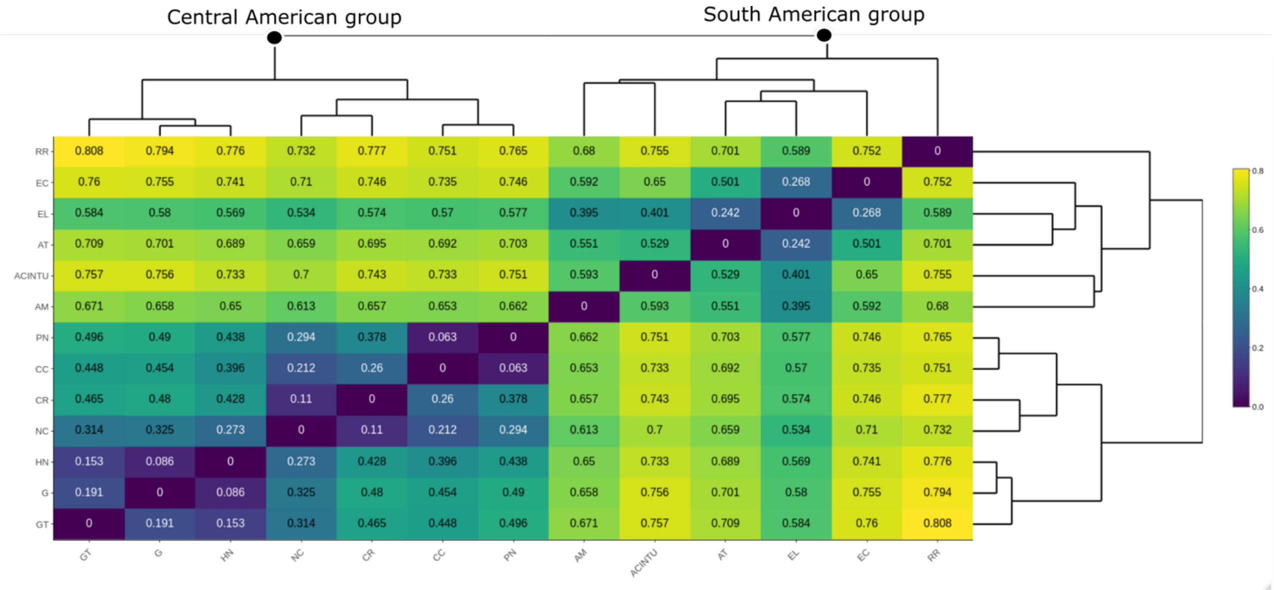

Supplementary Figure S3. Discriminant analysis of principal components (DAPC) for *A. aculeata* populations in Central America**. A.** Density plot based on 1523 makers showing the separation between the Mesoamerican, Costa Rican and, Panamanian groups. **B.** Scatter plot showing the separation between Mesoamerican, Costa Rican and, Panamanian subpopulations.

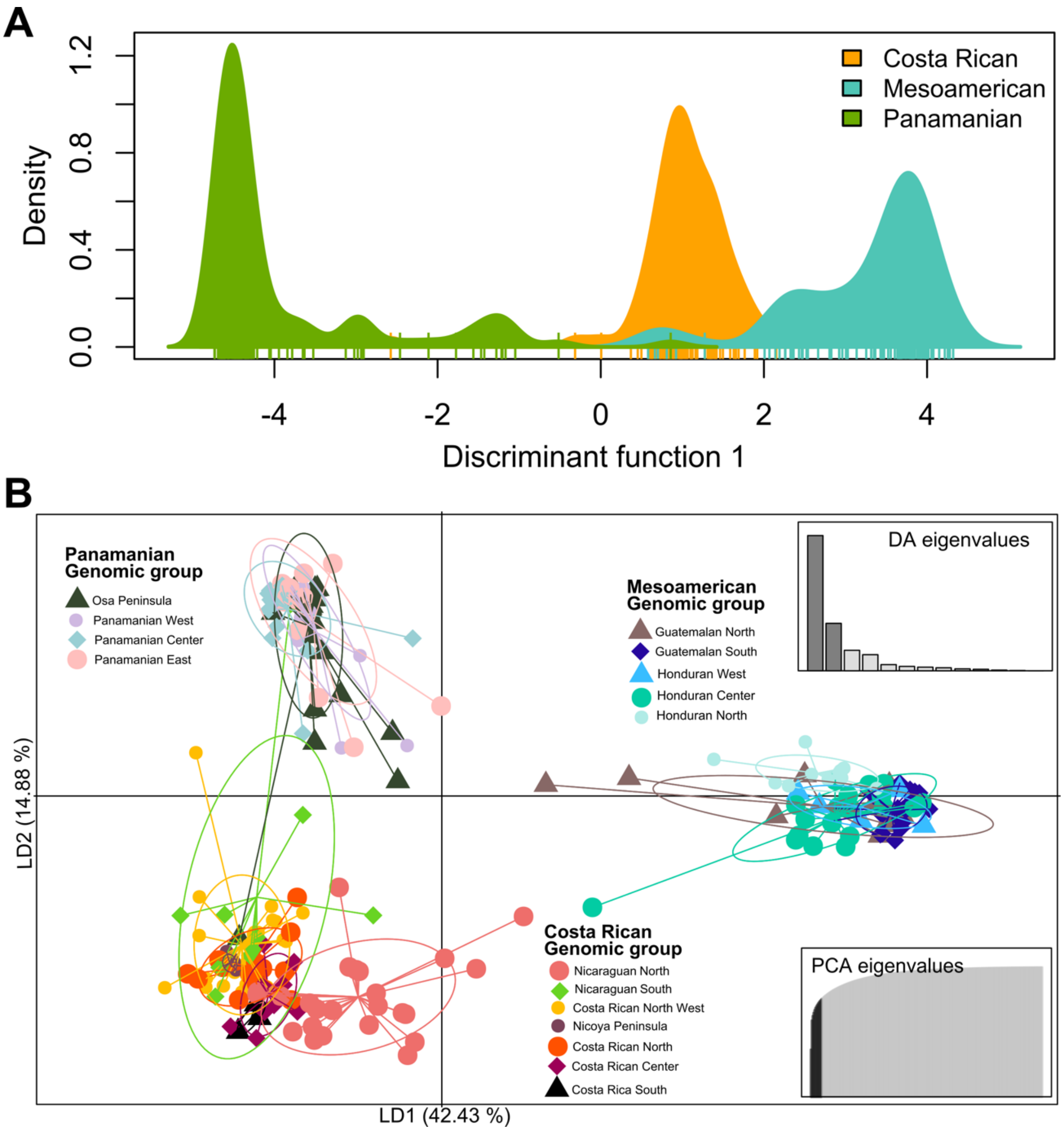

Supplementary Figure S4. Bar plots for the 3 major genetic clusters subpopulations in Central America. Admixture based on DAPC. Acronyms follow Table 1 Sub-regions.

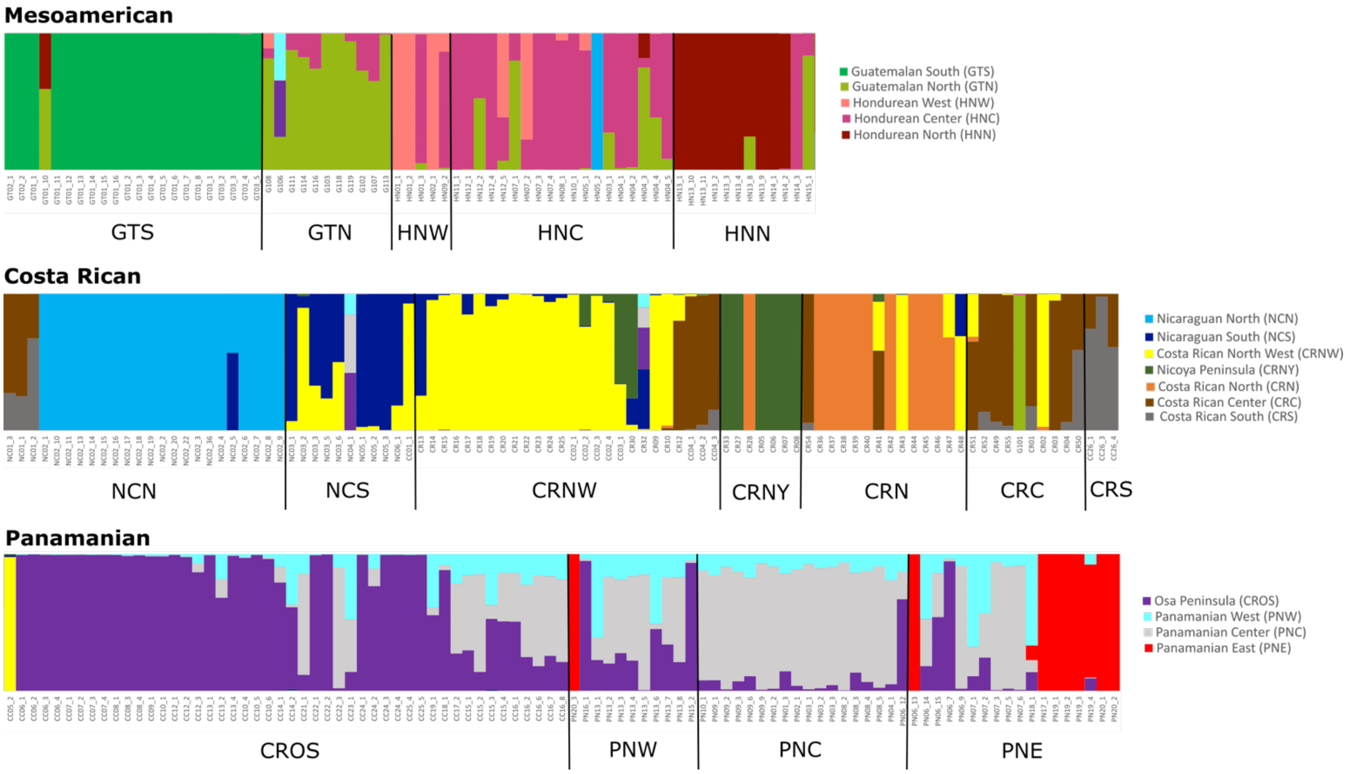

Supplement: Supplementary file 1 [file DataSheet1.pdf]
